# Mechanisms of lung damage in tuberculosis: implications for chronic obstructive pulmonary disease

**DOI:** 10.3389/fcimb.2023.1146571

**Published:** 2023-06-21

**Authors:** Alex Kayongo, Brian Nyiro, Trishul Siddharthan, Bruce Kirenga, William Checkley, Moses Lutaakome Joloba, Jerrold Ellner, Padmini Salgame

**Affiliations:** ^1^ Department of Medicine, Center for Emerging Pathogens, New Jersey Medical School, Rutgers, The State University of New Jersey, Newark, NJ, United States; ^2^ Department of Immunology and Molecular Biology, College of Health Sciences, Makerere University, Kampala, Uganda; ^3^ Makerere University College of Health Sciences, Lung Institute, Makerere University, Kampala, Uganda; ^4^ Division of Pulmonary and Critical Care Medicine, University of Miami, Miami, FL, United States; ^5^ Division of Pulmonary and Critical Care Medicine, Johns Hopkins University, Baltimore, MD, United States; ^6^ Center for Global Non-Communicable Disease Research and Training, School of Medicine, Johns Hopkins University, Baltimore, MD, United States

**Keywords:** Tuberculosis, COPD - chronic obstructive pulmonary disease, adaptive immunity, innate immunity, host-directed therapy (HDT)

## Abstract

Pulmonary tuberculosis is increasingly recognized as a risk factor for COPD. Severe lung function impairment has been reported in post-TB patients. Despite increasing evidence to support the association between TB and COPD, only a few studies describe the immunological basis of COPD among TB patients following successful treatment completion. In this review, we draw on well-elaborated *Mycobacterium tuberculosis*-induced immune mechanisms in the lungs to highlight shared mechanisms for COPD pathogenesis in the setting of tuberculosis disease. We further examine how such mechanisms could be exploited to guide COPD therapeutics.

## 1 Introduction

Chronic obstructive pulmonary disease (COPD) refers to chronic lung diseases characterized by slowly progressive irreversible airflow obstruction. Individuals diagnosed with COPD have varying degrees of chronic bronchitis, small airway obstruction, and emphysema (Cruz et al., 2007; Joint United Nations Programme on HA, 2011; Organization WH, 2015; Venkatesan, 2022). COPD is the third leading cause of death worldwide (Salvi, 2015; Venkatesan, 2022), with over 65 million people having moderate to severe COPD (Organization WH, 2015). It is one of the most common non-communicable diseases (NCDs), affecting over 329 million (Salvi, 2015). In 2012, COPD contributed to 6% of all total deaths globally, with more than 90% of mortalities occurring in low- and middle-income countries (LMICs) where effective strategies for prevention and control are not consistently implemented or accessible (Cruz et al., 2007). The prevalence of COPD, defined by spirometry, is 11.7% worldwide, and mortality shows an increasing trend (Adeloye et al., 2015).

Globally, over ten million people fell ill with TB disease in 2021, with over 1.4 million TB-related mortalities among HIV-negative individuals (Bagcchi, 2023). Men account for more TB cases than women (Organization WH, 2020; Bagcchi, 2023). Thirty high-TB-burden countries account for almost 90% of those who fall sick with the disease annually, with South-East Asia and Africa contributing to the most significant TB burden (Harding, 2020). Although cigarette smoking, exposure to pollutants, and HIV infection are predominant risk factors for COPD, pulmonary tuberculosis remains an under-recognized risk factor for developing COPD (Chakrabarti et al., 2007; Caballero et al., 2008; Allwood et al., 2014; Pefura-Yone et al., 2014; Byrne et al., 2015; Ngahane et al., 2016; Sarkar et al., 2017; Siddharthan et al., 2019; Kayongo et al., 2020; Byanova et al., 2021; Kamenar et al., 2021). Respiratory function is impaired in TB-induced COPD, characterized by significantly reduced forced vital capacity (FVC) and post-bronchodilator expiratory volume in 1 second (FEV_1_) compared to those with smoke-induced COPD (Anno and Tomashefski, 1955; Lee and Chang, 2003; Kamenar et al., 2021). Furthermore, the post-bronchodilator response is significantly reduced in tuberculosis-induced COPD compared to smoke-induced COPD, indicating the irreversible nature of airflow obstruction (Lee and Chang, 2003; Menezes et al., 2007). Tuberculosis-induced COPD risk is higher in males than females, with adjusted odds ratios of 4.0 and 1.7, respectively (Menezes et al., 2007). History of prior tuberculosis has been strongly associated with severe forms of COPD (Menezes et al., 2007; Kamenar et al., 2021). According to the Burden of Obstructive Lung Diseases (BOLD) study, a history of tuberculosis increases the risk of developing airflow obstruction in later life with an adjusted odds ratio of 2.5 (Amaral et al., 2015). The frequency and severity of airflow obstruction in pulmonary tuberculosis positively correlate with the number of episodes of tuberculosis (Hnizdo et al., 2000). Structural damage of the lungs increases with an increasing number of tuberculosis episodes and persists in many patients despite anti‐tuberculosis treatment (Plit et al., 1998; Hnizdo et al., 2000).

**Figure 1:**
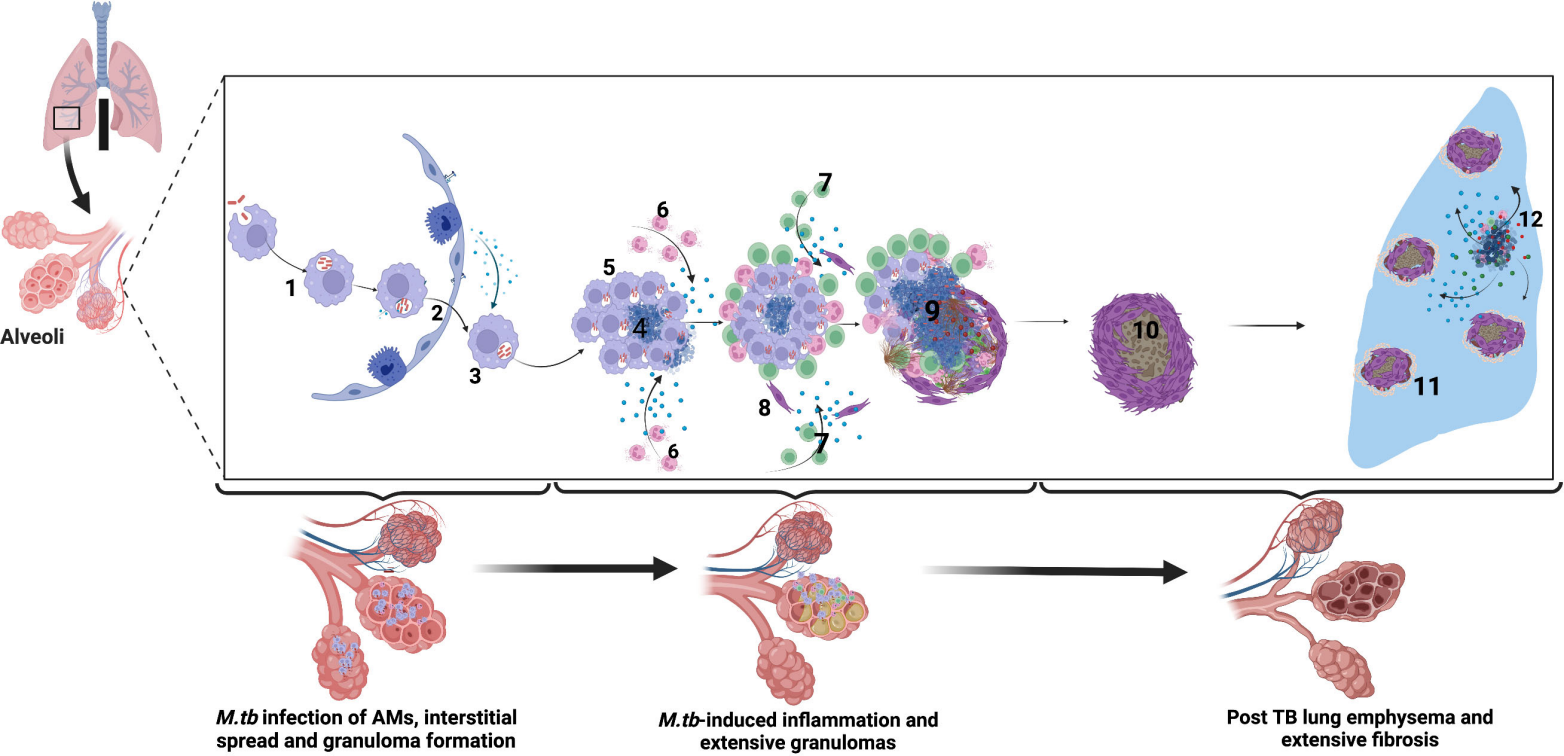
Immune-mediated mechanisms of TB-associated COPD. Alveolar macrophages engulf *M.tb* in the alveolar space. 2. Infected alveolar macrophages migrate from the alveolar space into the interstitium in an IL-1R-dependent manner. 3. M.tb replicate within alveolar macrophages. 4. *M.tb* induce infected macrophage apoptosis and expression of host lytic proteins in an ESX-1-dependent manner. 5. Newly recruited alveolar macrophages engulf infected cell debris. 6. Lung infiltrating neutrophils move by chemotaxis towards the growing granuloma, engulfing dying infected cells and killing bacteria through NETosis and release of lytic enzymes. 7. *M.tb*-specific T cells arrive at the granuloma and produce IFN-γ to enhance the microbicidal activity of alveolar macrophages. However, activated T cells are walled off from accessing the inner core of the granuloma, and their effect is dampened by the cytokine TGF*β*. 8. Alveolar macrophage necrosis leads to granuloma rupture and release of M.tb into the extracellular space. Subsequent induction of lytic proteins causes granuloma cavitation and release of Mtb into the airways. 9. In the post-TB stage following treatment, extensive lung fibrosis and emphysema reduces lung compliance and are observed as reduced lung function. 10-11. Extensive fibrosis and calcification further reduce lung compliance and worsens COPD. 12. Periodic insults such as bacterial, viral, and fungal infections and air pollution or smoking trigger periodic COPD exacerbations after that. Created with Biorender.com.

**Figure 2:**
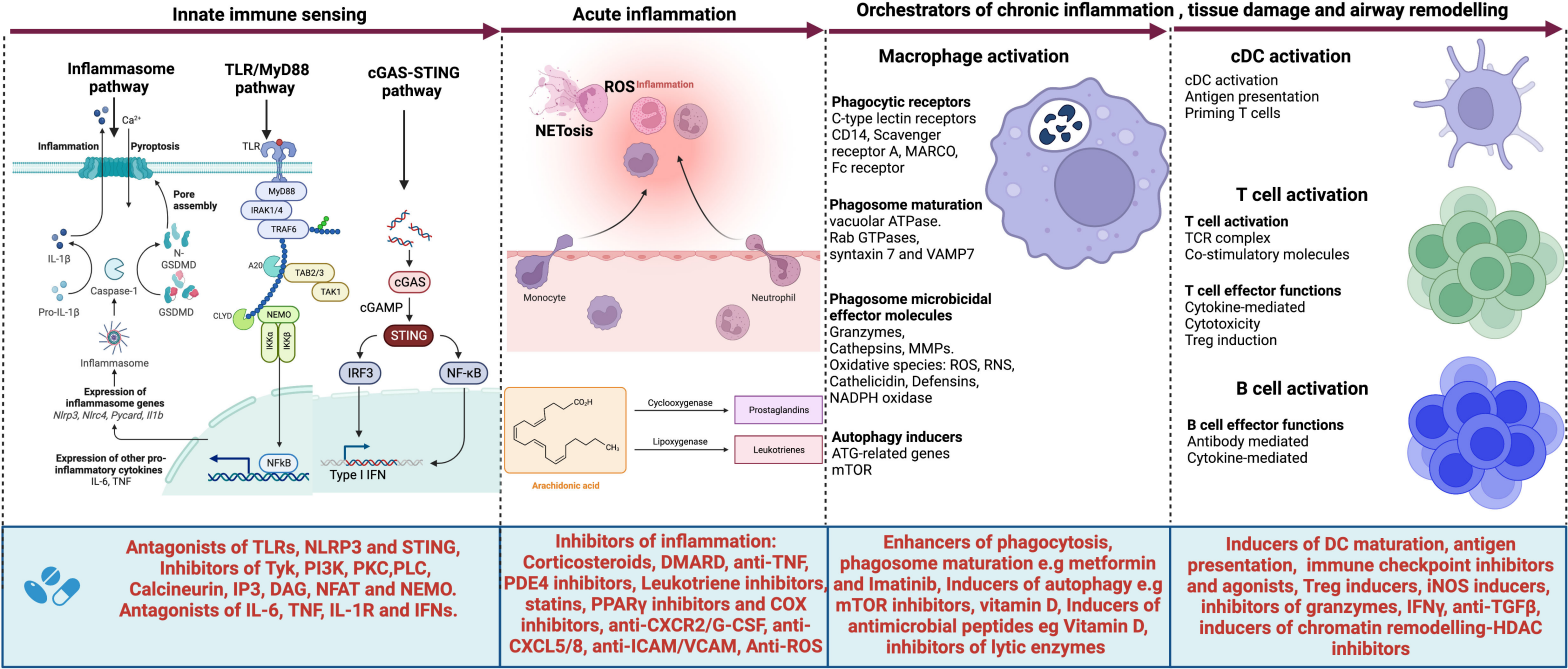
Targets for host-directed therapy (HDTs) for TB-associated COPD. A select list of immune-based therapies could offer benefits for patients with TB-associated COPD. Several agents have been developed or are under development that target (i) the innate immune sensors such as Toll-like receptors (TLRs) and their signaling pathway; (ii) the inflammasome activation pathway, their executioner Gasdermin D and pro-inflammatory cytokines IL-1β and IL-18; the cGAS-STING pathway and their effector cytokines, type I interferons as well as (iv) pro-inflammatory cytokines such as IL-6, TNF, IL1β and type I and II interferons. Such agents include antagonists of TLRs, NLRP3, and STING; inhibitors of the signaling molecules and enzymes such as tyrosine kinase (Tyk), PI3 kinase (PI3K), Phosphokinase C (PKCθ), phospholipase C (PLCγ), calcineurin, IP3, DAG, NFAT, and NEMO. Cytokine antagonists include anti-IL6, anti-TNF, IL-1R inhibitors, and inhibitors of type I interferons. Other agents target acute inflammation, such as corticosteroids, disease-modifying antirheumatic drugs (DMARDs), anti-TNF, Phosphodiesterase 4 (PDE4) inhibitors, Leukotriene BLT1-receptor antagonists, anti-TNF- therapies, TNFα-converting enzyme inhibitors, statins, PPARγ inhibitors, COX selective and non-selective inhibitors, TGFβ-1 receptor kinase antagonists, anti-IL-8 neutralizing antibody, CXCR2 inhibitors, CXCR3 antagonists, anti-Reactive Oxygen Species (ROS), anti-ICAM/VCAM and anti-CXCL5/8 and inhibitors. Other molecules target macrophage phagocytic receptors, autophagy, executioner machinery, and phagosome maturation. These include metformin, Imatinib as enhancers of phagosome maturation and autophagy induction, mammalian target of rapamycin (mTOR) inhibitors, Vitamin D, and inhibitors of lytic proteins and enzymes in phagolysosomes. cDCs, T and B cell activation also contribute molecules that orchestrate tissue damage. Such molecules can be targeted for therapeutic purposes. These include inducers of DC maturation like FLT3L, immune checkpoint inhibitors, inducers of Treg cells, inducers of iNOS, inhibitors of granzymes, anti-TGFβ, and finally, inhibitors of chromatin remodeling such as HDAC inhibitors. Created with Biorender.com.

Several predictors of COPD severity among tuberculosis patients have been reported, including smear‐positive disease, extensive pulmonary involvement before anti‐tuberculosis treatment, reduced radiographic improvement post‐treatment, and delay in initiating tuberculosis treatment (Chung et al., 2011). These factors imply that host-tuberculosis immune interactions in the lung microenvironment during active tuberculosis disease predominantly drive COPD pathogenesis. In this Review, we draw on well-described tuberculosis immune responses in the lung microenvironment to discuss immunological processes that could underlie the pathogenesis of COPD in the setting of tuberculosis disease (**Figure 1**). We further examine the extent to which such immunological processes could be exploited to guide tuberculosis-associated COPD therapeutics (**Figure 2**).

## 2 Organization of the immune system in the respiratory system

Distinct immune cell populations reside in the respiratory system, reflecting specialization along the tract to suit differing intensities of exposure to airborne antigens and airway microbiome in the transition between the upper and lower respiratory tract (Holt et al., 2008; Huffnagle et al., 2017). In conducting airways, the mucosal epithelial lining comprises ciliated cells interspersed with goblet cells, providing mucociliary clearance of inhaled antigens and locally secreted immunoglobulin A (IgA) (Holt et al., 2008; Hewitt and Lloyd, 2021). The mucosa contains dense networks of macrophages and dendritic cells (DCs) (Kopf et al., 2015). Airway macrophages are the most predominant cells residing on the mucosa and continuously sample airborne antigens (Cohen et al., 2018). The airway DC population comprises both myeloid DCs (mDCs) and plasmacytoid DCs (pDCs), with a predominance of mDCs (Kopf et al., 2015). A specialized group of DCs known for immune surveillance is strategically located within and directly below the mucosa (Holt et al., 2008). These DCs extend their protrusions into the airway mucosa, routinely sampling microbial antigens (Jahnsen et al., 2006). Another population of cells referred to as innate lymphoid cells (ILCs) resides within lymphoid tissue at airway branch points (Sonnenberg and Hepworth, 2019). They co-localize with CD4+T cells, DCs, and specialized stromal cells, which provide activating cytokines (Sonnenberg and Hepworth, 2019). Three ILC subsets closely mirroring the transcriptional and functional biology of CD4+T helper (Th) cells (i.e., ILC1, ILC2, and ILC3) have been described elsewhere (Sonnenberg and Hepworth, 2019).

T cells are also found in relatively high numbers in the mucosa and within the underlying lamina propria (Holt et al., 2008). Most intra-epithelial T cells express CD8, whereas CD4+T cells are more frequently found in the lamina propria (Holt et al., 2008). Both cell subtypes have effector and memory cell phenotypes defined by the expression of CD45RO (Holt et al., 2008). The submucosa also harbors B, mast, and plasma cells, mainly producing polymeric IgA (Brandtzaeg, 2015). In addition to their role in antibody production, airway B cells contribute to local antigen presentation. As described elsewhere (Holt et al., 2008), the airway mucosa also contains bronchial-associated lymphoid tissue (BALT), comprising discrete lymphoid-cell aggregates underlying a specialized epithelium, similar to Peyer’s patches in the gut. Under homeostasis, several immune cells, including interstitial macrophages, DCs, T cells, B cells, and mast cells, populate the lung parenchyma, whereas large numbers of T cells sequester in the lung parenchymal vascular bed (Holt et al., 2008). In the setting of inflammation, other cells, such as neutrophils and monocytes, infiltrate and predominate the lung parenchyma (Cohen et al., 2018). The contribution of bronchial epithelial cells, fibroblasts, and the extracellular matrix in airway immune response has been described elsewhere (Kitamura et al., 2011; Gao et al., 2015; Hewitt and Lloyd, 2021).

## 3 Immunological relationship between tuberculosis and COPD

Several authors have described the pathogenesis of COPD with emphasis on cigarette smoke, biomass exposure, and HIV infection (Hogg, 2004; Chung and Adcock, 2008; Churg et al., 2008; Rajendrasozhan et al., 2008; Atkinson et al., 2011; Milara et al., 2013; Pichavant et al., 2014; Agustí and Celli, 2017; Yoshida et al., 2019; Byanova et al., 2021). Other post-TB sequelae, such as bronchiectasis and bronchopulmonary aspergillosis, cannot be underestimated (Meghji et al., 2021). Whereas the role of innate and adaptive immune cells in COPD has been extensively described elsewhere (Barbu et al., 2011; Ezzati Givi et al., 2012; Craig et al., 2017; Le Rouzic et al., 2017; Ni and Dong, 2018), in this review, we describe how immune mechanisms in the setting of *Mycobacterium tuberculosis* infection intersect with known COPD-immune mechanisms and possibly contribute to immuno-pathology in TB-associated COPD. To date, literature describing the immunological relationship between tuberculosis and COPD remains limited (Radovic et al., 2011; Cao et al., 2012; Allwood et al., 2019; Chin et al., 2019; Singh et al., 2022). Recent evidence suggests that tuberculosis orchestrates chronic lung inflammation and tissue necrosis, with resultant airway fibrosis and remodeling observed in COPD (Radovic et al., 2011; Cao et al., 2012; Sarkar et al., 2017; Stek et al., 2018; Allwood et al., 2019; Chin et al., 2019; Singh et al., 2022). The immune response, particularly in the distal airways, is characterized by activation of alveolar macrophages, dendritic cells, innate lymphoid, and *γδ*-T cells, which promote recruitment and activation of neutrophils, monocytes, as well as cells of the adaptive immune response (B cells, Th1, Th17, and cytotoxic T-cells). Further interaction between immune cells and the airway epithelial cells, fibroblasts, and the extracellular matrix culminates into granuloma formation, which upon degeneration (caseation), orchestrates lung inflammation and tissue necrosis coupled with fibrosis and airway remodeling (Chung and Adcock, 2008). Although the immune mechanisms of lung parenchymal damage in tuberculosis have been extensively described and the role of host-directed therapy elucidated elsewhere (Ravimohan et al., 2018; Stek et al., 2018), this review focuses on the immunologic sequence of events in early versus late tuberculosis disease that orchestrate COPD immunopathology (**Figure 1**).

### 3.1 Mtb-induced innate immunity and implications on COPD

#### 3.1.1 Macrophages

Following infection of a host with *Mycobacterium tuberculosis (Mtb)* via inhalation of viable bacilli in exhaled droplets, bacilli are internalized by alveolar macrophages (AM) via phagocytosis (Pieters, 2008). Based on *in vitro* studies with various macrophage types, including AMs, viable bacilli have been shown to modify phagosome activity, preventing its maturation and fusion with lysosomes via mechanisms well-described elsewhere (Sturgill-Koszycki et al., 1994; Ferrari et al., 1999; Gatfield and Pieters, 2000; Fratti et al., 2003; Rosenberger and Finlay, 2003; Walburger et al., 2004; Nguyen and Pieters, 2005; Vergne et al., 2005; Rohde et al., 2007; Warner and Mizrahi, 2007; Pieters, 2008; Vandal et al., 2008; Sun et al., 2010; Abramovitch et al., 2011; O’Leary et al., 2011; Wong et al., 2011; Sullivan et al., 2012; Jamwal et al., 2016; Awuh and Flo, 2017; Queval et al., 2017; Buter et al., 2019). Some internalized bacilli perforate the phagosome membrane using the ESX-1 type III secretion system, escaping into the cytosolic space (Manzanillo et al., 2012; Augenstreich et al., 2017; Conrad et al., 2017; Queval et al., 2017; Wong, 2017). Therefore, by engaging several immune evasion strategies such as those described above (Hmama et al., 2015), *M.tb* bacilli successfully establish infection in the AMs dividing exponentially until the immune pressure contains the pathogen (Russell et al., 2009). Consequently, either localized sterilization of *M.tb* bacilli and mineralization of lesions into Ghon foci or extensive caseation and tissue necrosis occurs with lung damage and eventual release of *M.tb* bacilli into the airways (Russell et al., 2009). Although several authors have described macrophage activation in the context of the *M.tb* control (Kornfeld et al., 1999; Kaufmann, 2002; Nau et al., 2002; Flynn, 2004; Lin et al., 2007; Blumenthal et al., 2009; Moreira-Teixeira et al., 2016; BoseDasgupta and Pieters, 2018; Marakalala et al., 2018; Khan et al., 2019; Li et al., 2021; Mata et al., 2021; Thakur and Muniyappa, 2023), in this section, we elaborate on events surrounding macrophage activation in early and late *M.tb* infection, which orchestrate lung damage as observed in COPD.

In the early stages of *M.tb* infection, airways initially show increased infiltration with innate immune cells beneath the lamina propria, without any obvious pathological lesions (North and Jung, 2004; Russell et al., 2009). Until recently, the precise composition, pattern, and mechanisms underlying this immune cell infiltration within the airways in the early stages of *M.tb* infection have been largely speculated. However, evidence from the murine model shows that *M.tb* infection exclusively occurs in the AMs during the first week of infection (Cohen et al., 2018). In this period, *M.tb*-activated AMs infiltrate the lung interstitium in a MyD88/IL-1R-dependent manner to establish a focus of infection in the interstitium (Cohen et al., 2018; Lovey et al., 2022). This precedes chemotaxis and infiltration of the lung interstitium and the airways with neutrophils, monocyte, and lymphocytes. Cytokines produced by AMs play a critical role in attracting other immune cells into the primary area of insult to contain *M.tb* growth. For instance, TNF has been reported as a critical cytokine in the initial stages of granuloma formation and *M.tb* control (Kindler et al., 1989; Roach et al., 2002; Algood et al., 2005). This cytokine maintains the concentration gradient required for sustained cellular recruitment and retention into the growing granuloma (Roach et al., 2002). In the *tnfr* knock-out murine model, granuloma cellular organization is severely disrupted, resulting in an amorphous and necrotic structure that is poor at containing *M.tb* growth (Gil et al., 2006). However, as the infection progresses, too much TNF drives airway pathology, where the granulomatous response becomes too aggressive and causes extensive tissue destruction (Casadevall and Pirofski, 2003). Although the intention is usually to contain *M.tb* growth, this excessive response orchestrates airway damage and drives structural changes that severely impair lung function (Casadevall and Pirofski, 2003; Russell et al., 2009). Besides TNF, other pro-inflammatory cytokines such as IFN-γ, IL-12, IL-1β, and IL-18 not only drive *M.tb* killing but also sustain macrophage activation (Flynn, 2004; Koo et al., 2008; Fan et al., 2013; Yang et al., 2013; Moreira-Teixeira et al., 2016; Agarwal et al., 2021; Li et al., 2021), which sustains airway damage. What is currently unknown is the exact timing when such damage becomes irreversible to portray the pathology observed in COPD. However, from the natural history of COPD, repetitive insults accumulate over time and cause structural abnormalities with impaired lung function (Tantucci and Modina, 2012; Vestbo and Lange, 2016; Lange et al., 2021). In the setting of *M.tb*, airway insults most likely occur during the acute phase of inflammation and become sustained in the chronic phase. Unfortunately, multiple attempts at healing result in extensive fibrosis and airway remodeling, altering lung anatomy and distal airway mechanics (Dheda et al., 2005). Performing serial lung function tests in a murine infection model in a carefully designed experimental setting could assist in determining the specific time when irreversible damage occurs. Furthermore, conducting in-depth omics analyses of such changes would provide critical information about TB-associated COPD immunopathology.

Upon activation, AMs demonstrate plasticity, differentiating into either classical (M1) phenotype in the presence of specific cytokines such as IFN-γ or alternative (M2) phenotype in the presence of specific cytokines such as TGF-β and IL-10 (Khan et al., 2019; Locati et al., 2020; Ge et al., 2021; Ahmad et al., 2022; Arish and Naz, 2022). M1 phenotype is pro-inflammatory, expresses the *iNOS gene*, induces reactive oxygen (ROS)/nitrogen (Ernst, 2012) species (Ernst, 2012), and is highly specialized in phagolysosomal killing and containment of *M.tb* bacilli, while M2 is anti-inflammatory and aids in the clearance of debris at the down or resolution of inflammation (Kasmi and Stenmark, 2015; Marino et al., 2015; Arora et al., 2018; Shapouri-Moghaddam et al., 2018). M2 also drives tissue repair and regeneration (Kasmi and Stenmark, 2015; Marino et al., 2015; Arora et al., 2018; Shapouri-Moghaddam et al., 2018). Since macrophage polarization states affect the growth of *M.tb* bacilli in either a restrictive or permissive manner, a fine balance between M1 and M2 phenotypes ensures containment of the *M.tb* bacilli with minimal tissue damage (Refai et al., 2018; Chen et al., 2022). In chronic inflammation, excessive M1 activity orchestrates tissue damage. Macrophages unleash ROS and RNS species, mount oxidative stress, and cause bystander damage to surrounding tissue (Bogdan, 2001; Guirado et al., 2013). Other molecules, such as cathelicidins (Herr et al., 2007; Tecle et al., 2010), defensins (Ganz, 2003; Doss et al., 2010), cathepsins (Zavašnik-Bergant and Turk, 2006; Lecaille et al., 2008; Patel et al., 2018), matrix metalloproteases (Page-McCaw et al., 2007; Salgame, 2011; Houghton, 2015; Grzela et al., 2016), and S100 proteins (Donato et al., 2013; Kessel et al., 2013; Pouwels et al., 2014; Singh and Ali, 2022), drive connective tissue damage, fibrosis, and angiogenesis, as described elsewhere (Barnes et al., 2003; Guirado et al., 2013; Whitsett and Alenghat, 2015). Of particular importance are collagenases (MMP1 and MMP13), elastases (MMP12), and gelatinases (MMP2 and MMP9), which degrade underlying connective lung tissue, promoting tissue necrosis (Guirado et al., 2013). MMP-1 and MMP-8 levels correlate with airway tissue damage in TB patients, whereas MMP-14 promotes collagen degradation and regulates monocyte migration (Sathyamoorthy et al., 2015). Overall, existing evidence supports the role of these molecules in driving lung damage in TB. Following treatment with anti-TB drugs for at least two months, findings show a significant reduction in the serum levels of MMP-8 in TB patients with severe lung tissue damage (de Melo et al., 2019). Similarly, serum MMP-1, -2, -3, -9, and -12 levels are higher in TB patients with severe structural lung damage. These molecules decrease following successful treatment (Kumar et al., 2018). In emphysema and chronic bronchitis (which are forms of COPD), similar molecules mediate lung inflammation and promote the release of fibrogenic growth factors (Churg et al., 2012), suggesting that these molecules, in a way, contribute to TB-associated COPD. As previously noted, we currently do not know the exact time in the course of the disease when these molecules induce irreversible airway damage and tissue remodeling, hence warrant further investigation.

Besides the collateral damage from activated macrophages, the genesis and evolution of the TB granuloma (Russell, 2007; Russell et al., 2009; Ramakrishnan, 2012; Nunes-Alves et al., 2014; Kiran et al., 2016; Marakalala et al., 2016; Cadena et al., 2017; Cohen et al., 2022; McCaffrey et al., 2022), from its nascent form through caseous, fibrocaseous, and resolved forms as the infection progresses, plays a direct role in distorting the lung anatomy, as observed on chest X-ray films and CT scans of most post-TB patients (Stek et al., 2018). Similarly, evidence of impaired respiratory mechanics has been documented by spirometry among post-TB individuals (Amaral et al., 2015; Meghji et al., 2020; Mpagama et al., 2021; Ivanova et al., 2023). During the process of granuloma formation, activated AMs, as previously described, invade subtending epithelium and attract mononuclear cells from neighboring blood vessels through chemotaxis and form the cellular matrix of the early granuloma (Russell et al., 2009). As *M.tb* infection progresses, the newly recruited monocyte-derived macrophages engulf infected cell debris and contribute to primary granuloma expansion (Pagán and Ramakrishnan, 2015). Some of these newly infected macrophages exit the primary granuloma and establish secondary granulomas in distal tissues, spreading the infection further (Pagán and Ramakrishnan, 2015). Infected macrophages in the primary granuloma undergo necrotic cell death (Behar et al., 2011), majorly driven by pathogen-induced subversion of eicosanoid synthesis from prostaglandin E_2_ to Lipoxin A_4_ (LXA_4_) (Chen et al., 2006). This promotes lung inflammation, tissue destruction, and remodeling, favoring chronic lung damage. Other immune cells, such as neutrophils and lymphocytes, infiltrate TB granuloma and lung tissue (Cohen et al., 2018). Consequently, by the 3^rd^ week of infection, infected alveolar macrophage number plateaus while infected neutrophils and monocyte-derived macrophages increase drastically to become the most predominant cells in the granuloma (Russell et al., 2009; Cohen et al., 2018). In its nascent stage, the granuloma has a center of infected macrophages. As it evolves into its caseous form, these cells become enclosed by lipid-laden (foamy) macrophages, which drive caseous necrosis (Russell et al., 2009; Agarwal et al., 2021). In the fibrocaseous stage, the macrophages differentiate into multinucleated giant epithelioid cells surrounded by lymphocytes. A thick fibrous cuff forms outside the epithelioid cells at this stage, excluding lymphocytes from the granuloma core (Russell et al., 2009; Agarwal et al., 2021). In the resolved form, extensive fibrosis occurs, walling off this area. The bacillary load remains relatively constant in this period, establishing a state of latency in 90% of cases (Russell et al., 2009). In some scenarios, calcification occurs, giving rise to Ghon foci, where TB re-activation into active disease never occurs (Donald et al., 2021). However, in some situations, for instance, conditions associated with immunosuppression such as AIDS, *M.tb* re-activation occurs, driving active TB disease (Innes et al., 2009). For granulomas that show increasing accumulation of caseum in the core, extensive necrosis and breakdown occur, releasing viable bacilli into the airways to propagate the *M.tb* infection cycle (Russell et al., 2009). Despite a prolonged course of anti-tuberculosis therapy, the damage caused by extensive granulomatous disease and fibrosis in the airways is permanent (Pasipanodya et al., 2007). It persists and increases with future TB episodes (Plit et al., 1998; Hnizdo et al., 2000), resulting in an irreversible decline in pulmonary lung function and structural damage to the lung parenchyma observed among post-TB individuals (Hnizdo et al., 2000). Several studies have described this phenotype as pulmonary impairment after tuberculosis (PIAT) (Pasipanodya et al., 2007; Pasipanodya et al., 2010; Gandhi et al., 2016; Chushkin and Ots, 2017).

In a nutshell, airway macrophages play a critical role in orchestrating lung damage as a bystander effect in *M.tb* infection. Insults most likely occur during the acute phase of inflammation and become sustained in the chronic phase, as evidenced by increased uptake or intensity of fludeoxyglucose F18 on PET scans taken among TB patients a year after completion of anti-TB therapy compared to baseline (Malherbe et al., 2016). In such a setting, dysregulated healing results in extensive fibrosis and airway remodeling. Distorted lung anatomy and abnormal airway mechanics consequently ensue. Given the heterogeneity of immune cells infiltrating the airways during acute and chronic inflammation in *M.tb* infection, immunopathology is multifactorial. In the proceeding sections, we discuss how other immune cells drive lung damage in TB, highlighting similarities to COPD immunopathology.

#### 3.1.2 Neutrophils

Although earlier studies report neutrophilic infiltration into the sites of *M.tb* infection within hours of inoculation, the response depends on the route of *M.tb* exposure (Lowe et al., 2012). Several experimental infection animal models report neutrophilic infiltration into multiple perivascular sites an hour post-infection following intravenous *M.tb* inoculation (Long et al., 1931), while cutaneous infiltration in rabbit infection model following BCG inoculation occurs within 3 hours of infection, peaking at 12 hours (Shigenaga et al., 2001). In a murine model, neutrophils arrive at the site of dermal BCG inoculation within 4 hours, peaking at 24 hours post-intranasal or intrapleural BCG inoculation (Abadie et al., 2005). A sharp contrast exists when the exposure is aerosol or intratracheal. Following intratracheal BCG inoculation, a 1- to 2-week’ airway neutrophilic infiltration has been reported (Fulton et al., 2000). In a recently published murine infection model, airway neutrophilic infiltration occurs within the 1^st^ week and a few days post-infection, following interstitial localization of alveolar macrophages (Cohen et al., 2018), and as the infection progresses through the 3^rd^ week, neutrophils predominate the airways (Cohen et al., 2018). Few studies have investigated the timing of neutrophilic influx in early TB infection in humans. However, a massive influx of neutrophils into the airways occurs in individuals with established tuberculosis disease, associated with tissue necrosis (Ehlers and Schaible, 2013). Evidence shows that neutrophils accumulate in broncho-alveolar lavage fluid and sputum of individuals with active tuberculosis (Eum et al., 2010). Mechanistically, the inflammatory cytokine milieu in the airways activates the endothelium, increasing the expression of adhesion molecules, ICAM-1, E-selectin, and P-selectin, which results in a neutrophilic influx (Lowe et al., 2012). This influx is driven by several immune pathways. In the *IL-23/Th17* axis, alveolar macrophages, upon activation, produce IL-23, which activates mucosal Th17 cells to produce IL-17, associated with neutrophil recruitment into the airways (Sergejeva et al., 2005; Lockhart et al., 2006; Guirado et al., 2013). In a second pathway, activation of bronchial epithelial cells via TLR2 signaling promotes the secretion of CXCL5, a known ligand for CXCR2 receptors expressed on neutrophils. Consequently, neutrophils move into the airways following the CXCL5 chemokine concentration gradient (Nouailles et al., 2014). Early neutrophilic infiltration into the airways depends exclusively on CXCR2 and primarily on CXCL5 as genetic ablation of CXCL5 results in impaired neutrophil recruitment and reduced lung inflammation (Nouailles et al., 2014).

Whereas early neutrophilic inflammation plays a critical role in protecting against tuberculosis disease progression (Kisich et al., 2002; Dallenga and Schaible, 2016; Kroon et al., 2018), sustained neutrophilic inflammation harms the host. Following phagocytosis, neutrophils fail to neutralize viable *M.tb* bacilli. Instead, the highly oxidative state generated in the setting of *M.tb* infection drives neutrophils into necrotic versus apoptotic cell death (Dallenga and Schaible, 2016). Mechanistically, *M.tb* induces ESX-1-dependent neutrophilic necrosis driven by reactive oxygen species (ROS). Consequently, massive accumulation of infected and dying neutrophils further unleashes highly lytic and oxidative molecules which cause extensive tissue necrosis (Dallenga et al., 2017). Unfortunately, impaired dead cell removal drives further inflammation, even after *M.tb* sterilization. This greatly hinders the resolution of inflammation (Malherbe et al., 2016).

Infected and dying neutrophils mediate tissue damage via a number of pathways, as briefly explained in this section. The production of reactive oxygen species (ROS) during the oxidative burst exceeds cellular antioxidant capacity, damaging cellular structures, including lipids, protein, and DNA (Thannickal and Fanburg, 2000). This oxidative stress drives *M.tb*-induced tissue necrosis, further promoting tissue inflammation and damage (Sun et al., 2015; Dallenga et al., 2017). Secondly, NETosis, which involves the expulsion of DNA complexed with antimicrobial proteins into extracellular space to form neutrophil extracellular traps (NETs), contains *M.tb* bacilli. However, high levels of NETosis coupled with ineffective clearance of bacteria in tuberculosis pose pathological consequences in the lungs. The antimicrobial histones and peptides coating the NET-DNA are directly cytotoxic to lung tissue, and inadequate support of NETs causes deleterious inflammation of host tissue (Xu et al., 2009). NETs, particularly extracellular histones, also cause epithelial and endothelial cell death (Xu et al., 2011). Such NETosis-induced cell necrosis orchestrates lung inflammation and damage (Ramos-Kichik et al., 2009). *M.tb*-induced NETosis has also been associated with macrophage activation, which drives lung inflammation and tissue damage in TB disease, as previously described (Braian et al., 2013). Several human studies have validated the role of NETosis in lung tissue damage. A study reported high citrullinated H3, a standard NET marker in serum samples from tuberculosis patients with extensive pulmonary damage (de Melo et al., 2019), suggesting that NET formation is associated with severe lung tissue damage in TB patients. Similarly, NETosis was markedly increased in the airways of stable COPD patients in another study (Pedersen et al., 2015; Pedersen et al., 2018; Uddin et al., 2019). Extracellular DNA correlated with absolute neutrophil numbers in sputum and airway obstruction (Pedersen et al., 2018; Uddin et al., 2019). Other studies have reported similar correlations between NETosis and COPD (Obermayer et al., 2014; Grabcanovic-Musija et al., 2015; Uddin et al., 2019). NETosis and ROS in TB disease could contribute to irreversible lung damage, as observed in COPD, although mechanistic studies are needed to underpin the role of NETosis in COPD pathogenesis. Thirdly, the production of lytic molecules such as matrix metalloproteases (MMPs), cathepsins, S100 proteins, cathelicidins, and beta-defensins, as is the case for activated macrophages, also drive airway pathology (Pugin et al., 1999; Ong et al., 2015; Muefong and Sutherland, 2020). Finally, there is increasing evidence that activated neutrophils mediate tissue damage via cytokine production (Mantovani et al., 2011; Etna et al., 2014). High levels of IL-8, TNFα, and IL-1β secreted from activated neutrophils in TB disease correlate with enhanced neutrophil migratory capacity, airway recruitment, and heightened pro-inflammatory response in the lung environment (Muefong and Sutherland, 2020), which could contribute to the observed tissue necrosis. Previous studies have supported IL-8 as a well-known factor in the pathogenesis of COPD (Larsson, 2008; Zhang et al., 2011). IL-8 has been reported to recruit neutrophils in airways and induce increased MUC5AC and MUC5B mRNA expression in bronchial epithelial cells (Bautista et al., 2009). In addition, previous studies have demonstrated higher baseline levels of IL-8 expression in airway epithelial cells from patients with COPD compared to healthy controls (Schneider et al., 2010). TNFα has also been reported to promote tissue necrosis, lung fibrosis, and weight loss in COPD (De Godoy et al., 1996; Takabatake et al., 2000). Similarly, IL-1β has been demonstrated to promote lung inflammation, emphysema, and airway remodeling (Schneider et al., 2010). As COPD progresses in severity, airway neutrophilia increases and is associated with more significant airflow obstruction and accelerated lung function decline (Stănescu et al., 1996; O’Donnell et al., 2004).

#### 3.1.3 Innate lymphoid cells

Besides CXCL5 production by epithelial cells, alarmins (IL-25, IL-33, TSLP) (Vannella et al., 2016; Gupta et al., 2017; Roan et al., 2019) as well as cytokine production (IL-1β, IL-8, and G-CSF) (Wickremasinghe et al., 1999; Roan et al., 2019) results in further mobilization of innate lymphoid cells (ILCs). As elaborated in this section, these cells promote the recruitment of other immune cells, driving airway inflammation and tissue damage. Following infection, ILC2/3 cells rapidly accumulate in the airways, promoting the recruitment of monocyte-derived macrophages and neutrophils (Ardain et al., 2019). In a murine model, ILC3s accumulate in the airways early in the infection, followed by ILC2 later. As the infection progresses, the ILC3 number increases drastically, mirroring the expansion of alveolar macrophages. These cells precede the accumulation of monocytes and monocyte-derived macrophages in the airways. Interestingly, mice that lacked ILC3s exhibited a significant reduction in early AMs (Ardain et al., 2019). During *M.tb* infection, CXCR5 expression on circulating ILC3s is upregulated, and parallel increases in plasma levels of its ligand, CXCL13, have been observed in human studies. This implies that ILC3s migrate in response to CXCL13 concentration gradient (Ardain et al., 2019). Moreover, IL-23-dependent expansion of ILC3s in mice and the production of the cytokines IL-17 and IL-22 are critical inducers of lung CXCL13, promoting early mobilization of lung ILC3 and macrophages to initiate inflammation in an attempt to control tuberculosis. This, however, promotes tissue necrosis, consequently (Ardain et al., 2019). Similarly, ILC3-driven immunopathology has been reported in COPD. In a recent report, the frequency of natural cytotoxicity receptor-expressing ILC3 cells was increased in COPD lungs (Marashian et al., 2015). Other studies have shown that the frequency of ILC1 cells in patients with COPD correlates with disease severity and susceptibility to COPD exacerbations (Silver et al., 2016). In contrast, the combination of micro-CT analysis, histology, and gene expression profiling indicated that ILC1 signatures were enriched in centrilobular emphysema, suggesting that the alveolar destruction observed in COPD may be driven by ILC1s (Suzuki et al., 2017).

#### 3.1.3 Dendritic cells

In the airways, conventional dendritic cells (cDCs) exist as immature cells, residing beneath the mucosa for an extended period of time (Banchereau and Steinman, 1998). These cells continuously sample the mucosa via their dendrites’ extension in between the epithelial cells’ tight junctions (Artis, 2008). Immature cDCs partially mature to express CCR7, which allows them to home into draining lymphoid tissues, in which they interact with naive T cells (Geissmann et al., 2002). Upon contact with *M.tb*, immature DCs phagocytose the bacilli, become activated, and undergo differentiation from highly phagocytic into less phagocytic cells (Kapsenberg, 2003). At the onset of the inflammatory response, immature cDCs are highly represented at sites of *M.tb* infection and are specialized for antigen uptake (Sertl et al., 1986; Mihret, 2012). During differentiation, however, cDCs upregulate the expression of MHC class I and II molecules, CD40, CD54, CD58, and CD80, resulting in a cellular phenotype consistent with mature and activated DCs, excellent at antigen presentation (Mihret et al., 2011). Upon phagocytosis, the fate of antigen presentation to either CD4+ or CD8+ T cell depends on the intracellular *M.tb* routing (Burgdorf et al., 2007; Chatterjee et al., 2012; Cohn et al., 2013). Delivery of the pathogen cargo into the late endolysosomes results in a much quicker degradation and MHC class II antigenic loading, whereas delivery into early endosomes results in a slower degradation with prolonged MHC class I antigenic loading. Upon migration into draining lymphoid tissue, mature cDCs with class II antigen load prime naïve CD4+T cells, while those with class I antigen load prime CD8+T cells (Burgdorf et al., 2007; Chatterjee et al., 2012; Cohn et al., 2013). cDCs prime the adaptive immune system and contribute significantly to early, intermediate, and late cytokine production. During early *M.tb*-induced response, the release of early mediators of inflammation such as TNF-α, IL-1α, IL-1β, IFN-γ and chemokines such as CXCL5 and CXCL8 promotes the influx of additional monocytes, macrophages, and neutrophils into the airways (Etna et al., 2014). As previously described, these cells orchestrate inflammation, tissue necrosis, airway remodeling, and fibrosis. cDCs also phagocytose apoptotic bodies from dying neutrophils, and macrophages infected with *M.tb* bacilli, promoting cross-presentation via class I molecules. Notably, the sustained release of IL-12, IFN-γ, and IFN-β cytokines from activated DCs, particularly in the early stage of *M.tb* infection, drive early granuloma formation and containment of *M.tb* (Etna et al., 2014). However, excessive type I interferon production contributes to tissue damage in the chronic phase (LeibundGut-Landmann et al., 2007; Steinman and Banchereau, 2007). As elaborated in the next section, activated cDCs produce several molecules with Th-polarizing abilities. For instance, IL-12, IL-23, IL-27, and type I IFNs induce a Th1 phenotype, while MCP1 and OX40 ligand induce a Th2 phenotype (Kapsenberg, 2003). During chronic inflammation, DCs alternatively induce CD4+T cells to become suppressive by making IL-10 or differentiating into FOXP3+ CD4+T cells (Jonuleit et al., 2000; Luo et al., 2007). IL-10 counter-regulates inflammation and induces tissue healing. However, in chronic inflammation, it also drives fibrosis (Robb et al., 2016). Finally, activated cDCs also produce high levels of IL-4, IL-33, and TGF-β, which drive airway fibrosis (Mihret, 2012). Whereas the direct role of cDC in inducing airway damage in TB has not been described, the accumulation of mucosal cDCs in the small airways of COPD patients supports the role of such cells in the immunopathology of COPD (Demedts et al., 2007).

### 3.2 *M.tb* elicited adaptive immunity and COPD

Activating the adaptive immune response to *M.tb* restricts bacterial growth but rarely eliminates the bacilli (Ernst, 2012). Evidence suggests that adaptive immune responses contribute to airway pathology (Ernst, 2012) in TB disease as observed in COPD. This response depends predominantly on cell-mediated immunity because *M.tb* lives within macrophages; thus, effector T-cell responses are required to contain or kill the bacteria (Flynn and Chan, 2001). The role of B cells in controlling *M.tb* infection was still unknown until recently when it was demonstrated that antigen-specific B cells nurture and direct follicular-like T cells into lymphoid follicles to mediate *M.tb* control (Swanson et al., 2023).

#### 3.2.1 T lymphocytes

Within a week of *M.tb* infection, activated CD4+ and CD8+T cells migrate to the lung-draining lymph nodes (Feng et al., 1999; Ernst, 2012). By the end of a month, both cellular phenotypes have increased in the lung environment, demonstrating both effector and memory phenotypes (Feng et al., 1999). Half of these cells are CD69+ (Kornfeld et al., 1999; Refai et al., 2018; Chen et al., 2022), indicating that activated T cells migrate to the site of infection, interact with *M.tb*-infected macrophages and DCs through CD40L on CD4+ T cells and CD40 on macrophages or dendritic cells (Kalams and Walker, 1998; Clarke, 2000). The outcome was previously reported as enhanced antigen presentation and costimulatory activity resulting in the generation of robust CD4+ and CD8+T cells (Kalams and Walker, 1998; Clarke, 2000). However, recent studies show that IFNγ production by *M.tb*-specific CD4+T cells becomes locally restricted by the granuloma microenvironment despite ongoing antigen recognition through TGFβ dependent immune mechanisms (Gern et al., 2021). TNFα and type I IFNs orchestrate macrophage necrosis and release of *M.tb* into the extracellular space (Pagán and Ramakrishnan, 2015). Subsequent induction of several tissue proteases causes granuloma degeneration (caseation), orchestrating lung tissue inflammation, necrosis, fibrosis, and remodeling, resulting in cavitation and release of *M.tb* into the airways. Consequently, these changes gradually and irreversibly compromise lung function leading to obstructive airway pathology observed in COPD. In the presence of the necessary cytokine milieu (Cooper et al., 1995), various Th1 effector subtypes are produced, ranging from early activated cells producing only IL-2 to cells producing IFNγ and multifunctional cells expressing IL-2, IFNγ, and TNFα (Darrah et al., 2007; Cooper, 2009). The presence of these multifunctional cells is associated with protection and lung tissue inflammation and necrosis (Darrah et al., 2007; Cooper, 2009). In addition, cytolytic CD4+T cells secreting perforins and granulysin are produced, promoting necrosis of *M.tb*-infected macrophages. Multifunctional CD4+T cells have been reported frequently among tuberculosis patients (Winkler et al., 2005), individuals from endemic tuberculosis areas, and vaccinated infants (Scriba et al., 2008; Soares et al., 2008). The conditions optimal for multifunctional and cytolytic antigen-specific lymphocytes have yet to be fully defined in human studies; however, recent literature suggests the role of IL-12p70 in IFN-producing cells in maintaining this cellular profile (Cooper et al., 2007).

In addition to Th1 cells, tuberculosis-specific Th17 cells are induced following aerosol tuberculosis infection in mouse models; Th17 cellular immune response in the lung microenvironment depends on the secretion of IL-23 from activated macrophages (Khader et al., 2005). Also, gamma delta (γδ) T cells have been shown to produce IL-17 following a high-dose intranasal challenge with BCG in mouse models. A large portion of the IL-17 response in the mouse model is within the γδ T cell population (Lockhart et al., 2006). Interestingly, upon blockade of IL-17 secretion during a high-dose challenge, neutrophil recruitment into the lung microenvironment is significantly hindered, altering the subsequent inflammation and lung pathology (Umemura et al., 2007). The repeated challenge of tuberculosis-infected animals with tuberculosis antigen results in increased lung neutrophil infiltration and subsequent tissue necrosis (Taylor et al., 2003). Studies investigating the role of IL-17 in this enhanced pathology have found that increased tissue necrosis and neutrophilic infiltration is dependent on IL-23 and could be ablated by the delivery of anti-IL-17 antibody, suggesting that lung inflammation developing in response to chronic tuberculosis exposure depends on IL-23 and IL-17 (Darrah et al., 2007; Cooper, 2009). In human studies, CD4+ *M.tb*-specific IL-17- and IL-22-producing cells have been detected in individuals exposed to tuberculosis, although only IL-22 is seen in the lung (Scriba et al., 2008). Similarly, CD1- and MHC class I–restricted CD8+ T cells in the lungs, upon activation, produce cytotoxic granules which, upon degranulation on infected macrophages and dendritic cells, release perforins forming pores in cells and granulysin, killing both bacteria and infected macrophages (Ernst, 2012). In addition, CD8+T cells produce IFNγ, which potentiates macrophage function (Flynn and Chan, 2001). In summary, Th1 CD4+ and CD8+T cell activation in tuberculosis amplify macrophage activation and necrosis, while Th17 promotes neutrophil-mediated tissue necrosis, which drives lung inflammation, tissue damage, and remodeling.

#### 3.2.2 B lymphocytes

In addition to T cell-mediated immune responses to *M.tb*, naïve B cells also develop into activated plasma cells that secrete *M.tb*-specific antibodies and produce cytokines (Rao et al., 2015; Loxton, 2019). Antibodies regulate effector functions, including opsonization, antibody-dependent cellular cytotoxicity (ADCC), and antigen neutralization. Furthermore, activated B cells are effective antigen-presenting cells that respond to whole or partial pathogens, presenting processed antigen peptides through their MHC-II to CD4+T cells (Abbas et al., 2019). As a result, B-cells contribute to the induction of CD4+ T-cells responses to tuberculosis, providing early protection against infection and driving antibody-mediated phagocytosis in which they modify macrophage behavior (Phuah et al., 2012). B-cells also respond in a non-humoral manner when stimulated by *M.tb* to produce pro- and anti-inflammatory cytokines, including TNFα, IL-10, IL-1β, IL-17, and IL-21 (Du Plessis et al., 2016b; Du Plessis et al., 2016a). Plasma (memory B) cells predominantly drive IL-10, IL-21, and TNFα production (Du Plessis et al., 2016b; Du Plessis et al., 2016a). Through the production of IL-12, IFNγ, and TNFα, effector B cells drive Th1 responses potentiating classical macrophage activation and, consequently, lung inflammation, tissue damage, and fibrosis (Chan et al., 2014). Production of IL-4, IL-33, and TGF-β by effector B cells disrupts Th1 response and favors alternative macrophage activation, promoting fibroblast activation, tissue remodeling, and fibrosis (Zhang et al., 2012). Preliminary COPD studies have suggested B cells’ role in COPD pathogenesis. Lymphoid follicles consisting of B-cells and follicular dendritic cells (DCs) with adjacent T-cells have been reported in both parenchyma and bronchial walls of patients with smoke-induced emphysema (van der Strate et al., 2006). Similarly, reported oligoclonal antigen-specific B-cell reactions among current smokers have been published (Brandsma et al., 2009). Plasma cells derived from B-cell maturation occur in more significant numbers in sub-epithelial and submucosal glands in patients with COPD. Most of these B cells express IL-4 and IL-5 (Zhu et al., 2007), which promote mucous hypersecretion and stimulation of fibroblasts to produce TGFβ, promoting tissue remodeling. More research is needed to characterize *M.tb*-specific B cell responses in the context of COPD pathogenesis.

## 4 Other important mediators of tuberculosis-associated COPD

### 4.1 Bacterial genetics

Until recently, the role of bacterial genetics in *M.tb* clinical outcome was only associated with the host and environmental factors (Warner et al., 2015). With the advent of whole genome sequencing (WGS), seven human-adapted lineages were reported and associated with distinct virulence and transmissibility (Coscolla and Gagneux, 2014). The modern *M.tb* lineages (Lineages 2, 3, and 4) have been reported to be more virulent and transmissible than the ancient lineages (Lineages 1, 5, 6, and 7) (Wang et al., 2010; Sarkar et al., 2012). This heterogeneity in virulence and transmissibility has implications for the clinical outcome of the disease. For example, in a preclinical *M.tb* infection model, virulent Beijing strains cause higher bacillary loads, more lung damage, and earlier mortality compared to strains from other lineages (Ordway et al., 2007). Follow-up mechanistic studies have suggested that Beijing strains have enhanced capacity to inhibit protective immunity in the lungs through induction of higher levels of type-I interferons, lower levels of IL-12 and TNF-α, and reduced CD4/CD8+T-cell activation (Manca et al., 2001; Reed et al., 2004; Manca et al., 2005). However, other studies have indicated that the Beijing strains induce a more robust regulatory T-cell response than different strains, thereby down-regulating protective immunity (Ordway et al., 2007; Shang et al., 2011). Furthermore, a mouse infection model demonstrated that different *M.tb* transmission phenotypes are associated with distinct pulmonary pathologies (Verma et al., 2019; Lovey et al., 2022). High transmission *M.tb* strains (*M.tb*-HT) were related to necrotic caseating lung granulomas with great potential to cavitate, while low transmission strains (*M.tb*-LT) were associated with diffuse lung inflammation. Therefore, the distinct pulmonary pathologies related to the different *M.tb* genotypes could have implications for COPD. Literature examining the relationship between *M.tb* strains and COPD pathology is very limited. With recent evidence for pulmonary impairment after tuberculosis (PAIT) in the context of high versus low *M.tb* transmission strains, investigators are now examining the relationship between high vs. low transmission *M.tb* strains and COPD outcome phenotype. Follow-up human studies involving spirometry and radiological imaging in individuals diagnosed with high transmission versus low transmission strains will illuminate this relationship further.

## 5 The implication of immune pathways in guiding host-directed therapeutics for COPD

Immune pathways orchestrating lung tissue damage and fibrosis in tuberculosis or post-tuberculosis disease warrant a careful search into possibilities of using a select group of immune-based therapies optimal for patients diagnosed with tuberculosis-associated COPD (Barnes and Stockley, 2005). This section discusses a select list of immune-based therapies for tuberculosis, traditionally referred to as host-directed therapies (HDTs). Host-directed immune therapies have been extensively reviewed as potential adjuvants to the standard *M.tb* treatment and have demonstrated the potential to shorten TB treatment duration, curb emerging antimicrobial drug resistance, and reduce airway injury and fibrosis in post-TB patients (Wallis and Hafner, 2015; O’Connor et al., 2016; Wallis et al., 2016; Kolloli and Subbian, 2017; Yang, 2017; Du Plessis et al., 2018; Kaufmann et al., 2018). **Figure 2** summarizes the potential targets of host-directed therapy that could be used to treat TB-associated COPD. Below, we discuss a few host-directed therapies (HDTs) that have shown promise in clinical trials. Phosphodiesterase 4 (PDE4) inhibitor has shown promise in clinical investigations. In a 6-week trial, a PDE4 inhibitor improved lung function among individuals with moderate-to-severe COPD (Vestbo et al., 2009). In patients with advanced TB disease, PDE4 inhibitors improved FEV_1_ at six months post-treatment (Wallis et al., 2021). mTOR inhibitors have also been investigated as host-directed therapies in TB patients. In a similar study as described above, Everolimus, an mTOR inhibitor, enhanced the recovery of FEV_1_ at month 6 post-TB treatment (Wallis et al., 2021). Auranofin (oral gold salt), and ergocalciferol (vitamin D), tested in the same study, were reported ineffective (Wallis et al., 2021). Leukotriene BLT1-receptor antagonists had been developed to treat neutrophilic inflammation among COPD patients (Silbaugh et al., 2000; Beeh et al., 2003). The same antagonists also inhibit the neutrophil chemotactic activity of sputum obtained from COPD patients, indicating their potential clinical value (Silbaugh et al., 2000; Beeh et al., 2003). A human monoclonal anti-IL-8 neutralizing antibody has been tested in tuberculosis associated-COPD. Whereas it minimally reduced dyspnea scores, there was no statistically significant clinical improvement field (Mahler et al., 2004). CXCR2 inhibitors have proven particularly beneficial in COPD and are being tested in clinical trials (Widdowson et al., 2004). Also, CXCR3 antagonists, which inhibit the recruitment of CD8+ T-cells into airways, might be helpful (Saetta et al., 2002). Antioxidants such as N-acetyl cysteine, stable glutathione compounds, analogs of superoxide dismutase, and selenium-based drugs have also been developed for clinical use and selective inhibitors of iNOS (MacNee, 2000; Kharitonov and Barnes, 2003). Using non-selective signal transduction pathways inhibitors such as NF-kB and p38 MAPK inhibitors may result in immune suppression and impair host defense, worsening tuberculosis disease (Underwood et al., 2000; Castro et al., 2003). However, more selective PI3Kγ inhibitors may have relevant anti-inflammatory activity in COPD, and small molecule inhibitors of PI-3Kγ have been developed (Sasaki et al., 2000). In addition, Metformin and statins have been implicated as potential therapeutic agents for lung fibrosis (Wallis and Hafner, 2015; Sato et al., 2016; Tahir et al., 2020). Mechanistically, metformin enhances autophagy and improves mitochondrial bioenergetics (Bharath et al., 2020), whereas statins block 3-hydroxy-3-methylglutaryl coenzyme A (HMG CoA) reductase, the rate-limiting enzyme in the cholesterol biosynthesis pathway (Stancu and Sima, 2001).

In summary, whereas host-directed therapies have been extensively investigated as potential adjuvants to the standard *M.tb* treatment and have demonstrated the potential to shorten TB treatment duration, such evidence is lacking for COPD after tuberculosis. More importantly, the exact time when HDTs should be started in TB patients is unknown. Therefore, more studies are warranted testing the timing of administration and effectiveness of these molecules in the context of TB-associated COPD.

## 6 Conclusion

In summary, in this review article, we have described immune pathways that may drive the immunopathogenesis of tuberculosis-associated COPD and their implications in the management of tuberculosis associated-COPD. The immune responses are predominantly characterized by activation and migration of alveolar macrophages from alveolar spaces into the lung parenchyma, coupled with recruitment of neutrophils; monocytes derived macrophages, B-cells, innate lymphoid cells, Th1, Th17, and cytotoxic CD8+T-cells into lung microenvironment. Their interaction with airway epithelial cells, fibroblasts, and the extracellular matrix culminates in sustained lung inflammation, tissue necrosis, and airway remodeling. Therefore, these changes gradually compromise lung function. Using this knowledge, we can harness the latest developments in COPD immune therapies and utilize them to make host-directed therapeutic choices optimal for tuberculosis-associated COPD or prevent lung injury.

## Author contributions

AK: Conceptualization, Methodology, Original draft writing, and editing; BK, TS, BN, and WC: draft review and editing; ML: funding acquisition, draft review, and editing; JE: funding acquisition, draft review, and editing and PS: Conceptualization, Mentorship, Design, funding acquisition and drafting the manuscript for important intellectual content. All authors contributed to the article and approved the submitted version.
